# Cannabinoids for fibromyalgia pain: a critical review of recent studies (2015–2019)

**DOI:** 10.1186/s42238-020-00024-2

**Published:** 2020-05-29

**Authors:** Erinn C. Cameron, Samantha L. Hemingway

**Affiliations:** School of Psychology, Fielding Graduate University, 2020 De La Vina St, Santa Barbara, CA 93105 USA

**Keywords:** Fibromyalgia, Cannabinoids, Analgesic, Musculoskeletal pain, Chronic pain, Systematic review, Cannabis

## Abstract

**Introduction:**

Fibromyalgia is a chronic health condition characterized by widespread, severe musculoskeletal pain that affects an estimated 5–7% of the global population. Due to the highly comorbid nature of fibromyalgia, patients with the disorder often respond poorly to traditional pain treatments. Recent studies suggest that patient response may be more favorable to alternative analgesics, such as cannabis. However, the therapeutic potential of cannabis-based pain treatment for fibromyalgia remains unclear. The present study examined the most recent cannabis literature (2015–2019) and provides a critical review of current research on the safety and efficacy of medical cannabis treatments for fibromyalgia.

**Methods:**

We followed Preferred Reporting Items for Systematic Review and Meta-Analyses (PRISMA) guidelines in searching the PubMed and Medline databases using the search terms “cannabis + fibromyalgia” and then “cannabinoids + fibromyalgia.” Inclusion criteria were a) English language, b) published in peer review journals, c) published from 2015 to 2019, d) all study designs except for systematic reviews and meta-analyses, and e) all cannabis preparations.

**Results:**

The search identified five applicable studies involving 827 participants that used six different treatments. Review suggested several methodological problems pertaining to generalizability and validity.

**Conclusion:**

Although the critically reviewed studies superficially suggest that medical cannabis is a safe and effective treatment for fibromyalgia pain, serious methodological limitations prevent a definitive conclusion regarding the use of cannabinoids for pain management in fibromyalgia patients at this time.

Fibromyalgia is associated with widespread musculoskeletal pain that is commonly accompanied by additional symptoms such as fatigue, cognitive problems, mood disturbances, and problems with sleep (Clauw 2015; Palagini et al. 2016). In the absence of a definitive cure for fibromyalgia, treatment primarily focuses on symptom management and improving patient quality of life. Fibromyalgia is significantly more common in women and has a prevalence rate of 4% across Europe and North America with an approximated worldwide prevalence of 5–7% (Lan et al. 2016; Queiroz 2013). Additionally, some fibromyalgia patients experience psychological, social, and behavioral symptoms that further affect overall functioning and quality of life. While once considered a mysterious or unspecified condition of psychological or emotional origin, there is now empirical evidence, such as brain imaging studies, which have highlighted several biological underpinnings of many common fibromyalgia symptoms (Pomares et al. 2017; Schmidt-Wilcke and Diers 2017).

Pathophysiological symptoms of fibromyalgia include a sensitized or hyperactive central nervous system that is associated with an increased gain in pain and sensory processing (Clauw 2015; Queiroz 2013). Fibromyalgia can occur alone but is often comorbid with conditions such as irritable bowel syndrome (IBS) and tension headaches (Clauw 2015). It is also highly comorbid with a variety of autoimmune disorders characterized by inflammation, such as rheumatoid arthritis. When comorbidities are present, centralized pain can stem from various problems, making it hard to identify the precise source. Research has shown that fibromyalgia patients with comorbid disorders where the common pathway is pain are less likely to respond to typical pain treatments such as surgery or opioids (Clauw 2015).

Moreover, results have shown that, in some cases, fibromyalgia patients with multiple comorbid conditions that lead to pain respond well to centrally acting pharmacological therapies, such as cannabis (Fitzcharles et al. 2018; Phillips and Clauw 2013; Russo 2016; Walitt et al. 2016). However, there is conflicting evidence in the extant literature regarding the use of cannabis with fibromyalgia patients. Recent systematic reviews of randomized clinical trials (RCTs) examining the use of medical cannabis in the treatment of chronic pain presented limited and ambiguous evidence that cannabis exhibits analgesic properties for chronic pain resulting from fibromyalgia (Fitzcharles et al. 2018; Walitt et al. 2016). These results, in combination with rapidly changing national policies regarding cannabis use, highlight the need for an investigation of more recently published literature on this topic.

Minimal recent research has examined the use of cannabis for pain reduction in patients with fibromyalgia, with existing studies offering limited evidence for safety, efficacy, and tolerability. Moreover, there is a lack of methodological rigor among existing studies in this area. Additionally, comparative analysis of systematic data across relevant studies is challenging due to the low number of overall studies and the significant limitations, vast differences in methodology, and inconsistent results. Recent systematic reviews regarding the use of cannabis for fibromyalgia pain have been limited in scope, with only one identified study focusing solely on fibromyalgia patients (Walitt et al. 2016). Further, limitations include a lack of investigation of herbal preparations, with a majority of studies focusing on synthetic preparations. Also, few studies covered a broad range of study designs, focusing mainly on randomized clinical trials (RCTs). This review briefly summarizes the role of the endocannabinoid system in pain management with fibromyalgia patients and provides a critical review of selected studies from 2015 to 2019.

## Endocannabinoid system and pain management

Research has indicated an extensive endocannabinoid system in animals, comprised of systemic endogenous ligands and receptors with critical localization to nervous tissue in both the central nervous system and the immune system (Donvito et al. 2017; Fitzcharles et al. 2016; Silver 2019; Walker et al. 2019). The primary function of the endocannabinoid system in humans is to maintain homeostasis, which includes regulation of pain and inflammation (Fitzcharles et al. 2016; Guindon and Hohmann 2009; Silver 2019). The endocannabinoid system is integral to normal physiological functioning in humans and has been associated with the pathology of several neurological conditions (Russo 2016). In addition to endogenous endocannabinoids, exogenous molecules with cannabinoid properties, such as botanical cannabinoids, engage the endocannabinoid system (Silver 2019). Traditionally utilized as a plant preparation derived from *Cannabis sativa*, cannabinoids have been widely used throughout history for medicinal effects (Bridgeman and Abazia 2017).

Studies have indicated that cannabinoids play a role in the following physiological processes in human: neuronal plasticity (Azad 2004; Viveros et al. 2007), pain (Guindon and Hohmann 2009; Khasabova et al. 2008), anxiety (Gray et al. 2015), inflammation (Guindon and Hohmann 2009; Nakajima et al. 2006), neuro-inflammation (Malek et al. 2015), immune function (Cabral et al. 2015), and metabolic regulation (Jesudason and Wittert 2008). Additionally, research has shown that 62% of licensed medical cannabis users in the United States report chronic pain as their top reason for use (Boehnke et al. 2019). Other results have indicated that neuropathic and musculoskeletal pain are the two commonest reasons why individuals who suffer from chronic pain choose medical cannabis as an alternative analgesic (Fitzcharles et al. 2016; Vučković et al. 2018). This review will focus on musculoskeletal pain as a form of chronic pain experienced in conjunction with fibromyalgia.

## Cannabis use for symptom relief

An increasing number of women are reporting cannabis use for symptom relief, particularly for the relief of chronic pain associated with health problems that are more common in women, such as fibromyalgia (Finseth et al. 2015; McConnell et al. 2014; Ryan-Ibarra et al. 2015). While there are many cannabis treatments available, and recent research has indicated that cannabinoids of all types act simultaneously on multiple pain targets in the human body (Morales et al. 2017), existing evidence has been interpreted inconsistently. Currently, the efficacy, tolerability, and safety of cannabinoids for pain management with fibromyalgia patients is highly questionable. Additionally, available research in this area has many limitations, including the lack of clinical trials, problems with internal and external validity, low sample sizes, short treatment duration, lack of generalizability, contradicting results, and modest observable effects. Our review covers gaps in the literature by reviewing studies from the past 5 years only, thereby providing the most recent coverage. Further, we included a broader range of studies such as comparative studies, observation studies and retrospective reviews, whereas the majority of past reviews only included RCTs.

## Method

The Preferred Reporting Items for Systematic Review and Meta-Analyses (PRISMA) was used for this review (Moher et al. 2009). We identified Medline and PubMed as databases for our research. A search was conducted in October 2019 using the keywords “cannabis + fibromyalgia” and then “cannabinoids + fibromyalgia.” Specific inclusion criteria were as follows: a) English language, b) published in peer review journals, c) published from 2015 to 2019, d) RCTs, comparative studies, observational studies, or retrospective reviews, and e) all cannabis preparations. Systematic reviews, meta-analyses, and literature older than 2015 were not included in this review.

## Results

**Fig. 1:**
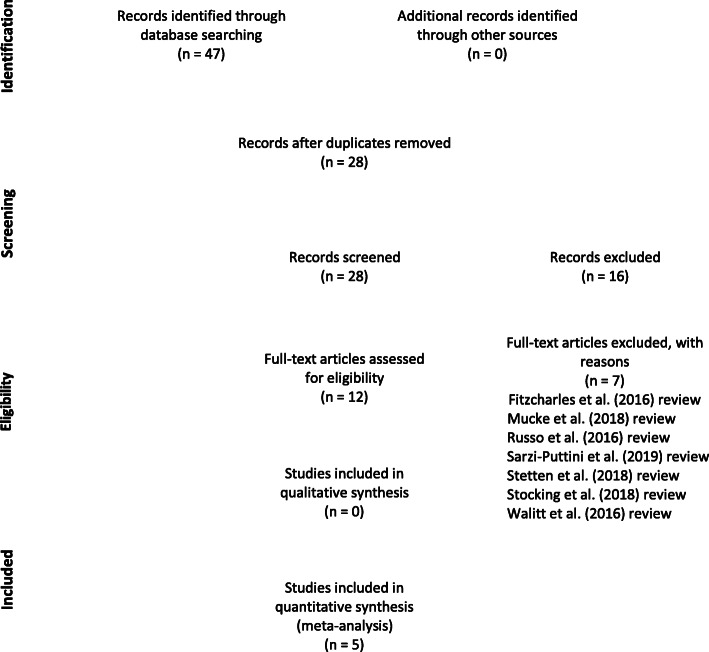
Literature search process

The initial search returned a total of 47 articles. The removal of duplicates resulted in 28 articles. All authors reviewed abstracts to determine relevance to the review topic. After eliminating articles that were not in English and those that did not meet study criteria, only five articles were deemed relevant; four from Israel and one from the Netherlands (Habib and Artul 2018; Habib and Avisar 2018; Sagy et al. 2019; Van de Donk et al. 2019; Yassin et al. 2019). Studies that discussed the role of cannabinoids in conditions other than fibromyalgia were included only when the study also referenced fibromyalgia. The reference sections of the selected articles were also reviewed for additional studies, although no additional studies were included. The goal was to critically analyze only the most current studies regarding cannabinoids in the treatment of chronic pain in fibromyalgia patients. Figure 1 presents a flow chart outlining our literature search process. See Table 1 for a summary of selected studies.

**Table 1:** Summary of selected studies

Authors	Study type	N	Duration	Assessment	Agent & dose	Concurrent analgesics	Delivery method	Adverse side effects	Results
Habib and Avisar (2018)	Online self-report survey	383 FM patients; 85% female	N/A	Self-report survey	31.4 ± 16.3 g; Self-report 3+ strains used (not specified); whole plant material and oil extract	15%	Inhalation (smoking) (80%); oral administration (oil) (5%); vaporization (15%)	Eye & throat irritation	Better sleep (94%); Decreased pain (93%)
Habib and Artul (2018)	Retrospective review	26 FM patients; 73% female	N/A	FIQR; self-report survey	26 ± 8.3 g per month; species not specified; whole plant material and oil extract	59%	Inhalation (smoking) (58%); vaporization (23%); both (14%); inhalation (smoking) & oral administration (oil) (8%)	Dry mouth (27%); red eyes (27%); hunger (15%)	Increased capacity for work (46%)
Sagy et al. (2019)	Prospective observational study	367 FM patients; 82% female	6 months	Pain intensity assessment; QOL	Varied gradual titration; 14 strains (not specified); whole plant material and oil extract	Y	Inhalation (smoking); oral administration (oil)	Mild dizziness (7.9%); Dry mouth (6.7%); Gastrointestinal symptoms (5.4%)	Better sleep (73.4%); decreased depression (80.8%); Pain reduced (44%)
Van de Donk et al. (2019)	Experimental randomized study	20 FM patients; 100% female	3 h	Pressure and electrical pain thresholds; spontaneous pain scores	Bedrocan (22.4 mg); Bedrolite (18.4 mg); Bediol (13.4 mg); Placebo	N	Vaporization (100%)	Drug high Bedrocam (80%); coughing (70%); nausea (15%); dizzy (15%); sore throat (10%)	Bediol 30% reduction in pain scores; THC correlated with an increase in pain threshold
Yassin et al. (2019)	Observational cross-over study	31 FM patients; 90% female	3–6 months	FIQR; VAS; ODI; SF-12; ROM	20 g; strain not specified; whole plant material	Y	Inhalation (smoking); vaporization	Red eyes (90%); Constipation (50%); loss of appetite (26%)	Decreased pain intensity; increased ROM

*FM* Fibromyalgia, *FIQR* Revised Fibromyalgia Impact Questionnaire, *QOL* Quality of Life, *VAS* Visual Analogue Scale, *ODI* Oswestry Disability Index, *SF-12* Short Form Health Survey, *ROM* Range of Motion

## Critical review of selected studies

Several limitations and methodological concerns were repeated across all five selected studies, indicating insufficient internal and external validity. The selected studies all assessed pharmaceutical cannabinoid products as therapeutic agents; however, each study examined a different cannabis preparation. Route of administration (ROA) also varied across studies and included delivery methods of smoking, inhalation via vaporization, and oral administration (oil drops). Dosage amount, treatment strategy and duration, diagnostic criteria, inclusions criteria, and baseline considerations were also inconsistent across selected studies. The inconsistencies in both methodological design and results across existing studies make the establishment of a solid foundation of empirical evidence challenging.

Additionally, since cultural considerations are not as relevant when determining the biological effectiveness of cannabinoids for pain management within the human species, the lack of broad cultural diversity among study participants was not considered a limitation in the selected studies. Moreover, since studies have shown that fibromyalgia has a high female:male ratio, it was not surprising that the majority of study participants across all selected studies were female. However, research has shown that there are significant biological differences between males and females regarding all areas of cannabis use, including addiction potential and outcomes (Cuttler et al. 2016; Fairman 2016; Hernandez-Avila et al. 2004; Kerridge et al. 2018; Schepis et al. 2011). These results indicate that gender is a significant consideration when considering the generalizability of cannabis studies of any kind. For cannabis to be recommended as a safe and effective treatment for chronic pain symptoms in fibromyalgia patients, studies must implement appropriate methodological design so that standardization of study protocol, treatment compounds, and regimens can be established (Sagy et al. 2019).

### Route of administration

Studies have indicated that ROA appears to have a distinct influence on health outcomes from cannabis use, with some ROAs having a higher instance of adverse health effects than others (Aston et al. 2019; Russo 2016). The most common ROAs include smoking, inhalation via vaporization, oral administration, and transdermal (Bridgeman and Abazia 2017). As revealed in multiple systematic reviews, respiratory problems such as coughing and wheezing, increased phlegm production, reduced pulmonary function, bronchodilation, and chronic bronchitis have been associated with smoking cannabis (Gates et al. 2014; Ghasemiesfe et al. 2018; Martinasek et al. 2016; Tashkin 2014). Additionally, researchers have noted that daily cannabis use via inhalation may cause adverse pulmonary effects over an extended period (Nugent et al. 2017). Habib and Artul (2018) noted that patients whose primary ROA was smoking were more likely to report transient adverse side effects of dry mouth and redness of the eye. Russo (2016) noted that smoking is undesirable for therapeutic application of cannabis, particularly with patients who have chronic conditions.

Widely understood to be a safer alternative, recent studies suggest that vaporization of the cannabis flower may provide distinct therapeutic advantages as compared to other ROAs (Aston et al. 2019; Lanz et al. 2016; Russo 2016). Vaporization of the botanical cannabis flower should not be confused with the use of the e-cigarette (vaping), which heats a concentrated form of cannabis oil to a high temperature and has recently been implicated in vaping-related acute lung injury (VpRALI) and adverse effects on the cardiovascular system (Fonseca Fuentes et al. 2019; Qasim et al. 2017). Only one of the studies selected for this review utilized vaporization in 100% of study participants (Van de Donk et al. 2019).

While there was little continuity across selected studies regarding ROA, all but one study (Van de Donk et al. 2019) utilized ROAs for which safety and efficacy are not well-supported in the extant literature. Sagy et al. (2019) reported using smoked joints, oil, or a combination of the two methods, noting that the choice was made by the study participant and was not tracked by the researchers. Habib and Avisar (2018), relying on self-report data only, reported that 80% of participants smoked cannabis in some form, 15% used vaporization, and 5% used oil. Habib and Artul (2018), also through self-report measures, noted that 58% of participants smoked, 23% vaporized, 14% combined vaporization and smoking, and 8% combined smoking and oil. Yassin et al. (2019) reported that study participants either smoked joints or used vaporization, but that information was not tracked across participants. Van de Donk et al. (2019) utilized vaporization in 100% of study participants, which is currently the ROA with the most supporting empirical evidence for safety. The utilization of ROAs for which safety and efficacy are not supported by empirical evidence is highly concerning, especially given the 60% increase in worldwide cannabis use over the past decade (United Nations Office of Drugs and Crime (UNODC) 2019).

Unfortunately, there is a paucity of systematic data for comparative assessments regarding ROAs and the therapeutic use of cannabis (Russell et al. 2018). Additional research on ROAs is needed to establish a baseline for all further treatments and studies, lending increased validity to future research regarding the safety, efficacy, and tolerability of cannabis for all conditions. ROAs with a reliable and measurable onset that allows dose titration without causing pulmonary or other damage while resulting in effective symptom relief are needed. Additionally, cannabis drug formulations should be precisely biochemically defined with mandated consistency across producers.

### Agents assessed in selected studies

A significant limitation to establishing the utility of cannabis in fibromyalgia patients is the large variability in the examination of different types of cannabinoids both within and across studies. Botanical cannabis products were assessed as therapeutic agents in each of the selected studies; however, each study examined a different cannabis preparation. Botanical cannabinoids are plant-based with a varied composition that is challenging to determine as it varies even within parts of the same plant (Silver 2019).

Of the many cannabinoids identified in cannabis, tetrahydrocannabinol (THC) and cannabidiol (CBD) represent two principal components (Madras 2019). THC, the major psychoactive component of cannabis, has been shown to influence pain, appetite, orientation, and mood. In contrast, CBD, a non-psychoactive component of cannabis products, has anti-inflammatory, anti-anxiety, and analgesic effects (Stith et al. 2019). Although THC and CBD both elicit pharmacological effects through interactions with cannabinoid CB1 and CB2 receptors, THC is a receptor partial agonist, while CBD is a negative allosteric modulator of the CB1 receptor (Hryhorowicz et al. 2019). Due to their varying properties and molecular interactions, the relative proportion of THC to CBD in cannabis products determines the type of effect, pharmacokinetics, and adverse effects associated with each unique strain (Madras 2019).

Therefore, a key aspect in determining the efficacy and safety of cannabis agents needs to involve not only tracking the precise agent used with patient outcomes but also noting the ratio of THC to CBD in each dosage. However, identification and isolation of cannabinoids across products is challenging due to the lack of available information in this area. Moreover, as previously noted, research has shown that the mechanism of entry into the human body of different agents plays a role in efficacy and safety. Future research is needed to ascertain the most appropriate ROA for each agent, which is currently difficult due to rapidly changing cannabis-related technology.

#### Agent characteristics and dosage

Evidence highlighting the efficacy of cannabis in the treatment of chronic pain for fibromyalgia patients will not have acceptable validity if the type, strain, and dosage is not carefully tracked. Further, correlations between assessed outcomes and specific types of cannabis cannot be accurately determined if dosage and strain are not carefully tracked alongside outcomes. In the selected studies, Sagy et al. (2019) utilized 14 unspecified strains of cannabis that had been approved by the Israeli Ministry of Health with unverified self-reported dosages. Yassin et al. (2019) assessed the effects of unspecified strains of medical cannabis (1:4 THC: CBD) with a set dosage of 20 g from producers that had also been approved by the Israeli Ministry of Health.

Habib and Avisar (2018) did not document specific type or strain and study participants self-reported using as many as three or more unspecified and unverified strains of cannabis throughout the study. Additionally, Habib and Artul (2018) noted that only licensed cannabis (by the Israeli government) was used, but also did not document type, strain, or provide a description. Further, Van de Donk et al. (2019) assessed the characteristics and effects of cultivated cannabis substances administered in controlled dosages: Bedrocan (22.4 mg THC, < 1 mg CBD), Bedrolite (18.4 mg CBD, < 1 mg THC), and Bediol (13.4 mg THC, 17.8 mg CBD). Van de Donk et al. (2019) were the only researchers across the selected studies that precisely tracked agent type and dosage with outcome across each participant. Both Yassin et al. (2019) and Van de Donk et al. (2019) noted the ratio of THC to CBD in each dosage, which is an additional methodological practice that should be followed in all such studies. Official monitoring of cannabis type, strain, composition, and dosage, as well as verified dosage adherence, are critical aspects of study validity.

### Participant characteristics

There was a high level of demographic variety across all five selected studies. However, minimal effort was made to minimize bias by ensuring that groups were appropriately comparable at baseline for demographic and other key factors. While diversity would generally lead to higher generalizability, when assessing cannabis use for fibromyalgia, variability across participants is not as desirable. For example, cannabis use and efficacy are highly affected by gender (Calakos et al. 2017), and while one of the selected studies controlled for gender (Van de Donk et al. 2019), the others did not. Regarding patient characteristics, methodological problems across studies included lack of consideration for comorbidities, diagnostic consistency, concurrent analgesic use, history and tolerance of cannabis use, and cross-drug tolerance.

#### Gender

While gender-specific studies on cannabis use itself are few, those available have shown that gender differences do exist, most notably that males are more likely to use cannabis medicinally and that females are quicker to become addicted (Cuttler et al. 2016; Fairman 2016; Kerridge et al. 2018). Research has also shown that females are more sensitive to the subjective effects of cannabis, which can lead to an increased vulnerability for developing cannabis use disorder (Cooper and Haney 2016). Additionally, studies have indicated that males are not as sensitive as females to the adverse effects of cannabis on the brain (Wiers et al. 2016). Considering these results, studies comparing the efficacy, safety, and tolerability of cannabis use in fibromyalgia patients should control for gender, thereby increasing study validity. Due to the differing biological mechanisms and implications for differences in endocannabinoid functioning in males and females, study results are not likely generalizable across genders.

As previously noted, the majority of participants across the selected studies were female; 85% (Habib and Avisar 2018; Habib and Artul 2018), 82% (Sagy et al. 2019), 90% (Yassin et al. 2019), and 100% (Van de Donk et al. 2019). The high rate of female participants across studies might be expected due to an overall higher prevalence of fibromyalgia diagnosis in females. However, the three studies that included both males and females failed to take into consideration the differences in cannabis use patterns, propensity for addiction, and the biological mechanisms of cannabis interaction between sexes. Further, four of the five studies did not consider controlling for gender when analyzing and reporting results. Additionally, there is little research highlighting the differences in efficacy and safety of agent, strain, and ROA across genders. More research is needed in order to assess the generalizability of cannabis efficacy results across genders. Of the selected studies, the results of Van de Donk et al. (2019) (100% female) are the most generalizable across diverse female populations.

#### History of cannabis use

Studies have indicated that past and concurrent cannabis use influences the efficacy, safety, and tolerance of any form of concurrent analgesic use in chronic pain patients across diverse diagnoses (Salottolo et al. 2018). For example, research has shown that cannabis users with both neuropathic and musculoskeletal pain due to injury experience more inadequate pain control with standard analgesics and cannabis as compared to non-cannabis users with the same type of injuries, which may lead to higher opioid use when cannabis patients engage in concurrent usage patterns (Salottolo et al. 2018). Furthermore, studies have shown that recreational cannabis users have overall lower mean pain ratings than non-users (Yanes 2019). Additionally, research results suggest that the severity of adverse effects among current cannabis users is significantly lower than that of past cannabis users (6 months or more) or those who never had before used cannabis in any form (naïve users) (Ware et al. 2015). Moreover, studies have indicated that chronic cannabis use affects the pain response to injury and often results in increased opioid use (Yanes 2019). Given these results, confirming past and concurrent cannabis use is a critical aspect of study design for this area of research.

Yassin et al. (2019) did not screen participants for past or concurrent cannabis use. Van de Donk et al. (2019) excluded individuals who indicated recent cannabis use but did not indicate a timeframe or operationalize “recent” use. Sagy et al. (2019) asked participants about concurrent use of “other medications,” including recreational cannabis, but screening for past medical cannabis use was not indicated. Two studies (Habib and Artul 2018; Habib and Avisar 2018) assessed current but not past cannabis use. Only one of the selected studies (Van de Donk et al. 2019) implemented official drug screening tests to verify self-reported cannabis and concurrent use of other substances. Empirically sound conclusions regarding the efficacy and safety of cannabis for pain management in fibromyalgia patients lack validity when study design does not account for the effects of past and concurrent cannabis use.

#### Cross-drug tolerance

Studies have indicated that cross-drug tolerance is an important factor when assessing the efficacy of analgesics for pain management as cross-drug tolerance varies widely between individuals and substance interactions (Askay et al. 2009; Boehnke et al. 2019). In the selected studies, Yassin et al. (2019) tracked information regarding past opioid use, only including participants for whom opioids had not been successful for pain management. However, the researchers did not account for any other concurrent medications. Sagy et al. (2019) asked participants about the concurrent use of other medications; however, this aspect was not controlled for in the study design or analyses. Van de Donk et al. (2019) excluded patients who tested positive for cocaine, amphetamines, cannabinoids, phencyclidine, methadone, benzodiazepines, tricyclic antidepressants, and barbiturates. Habib and Avisar (2018) did not assess concurrent drug use of any kind, while Habib and Artul (2018) asked participants to document analgesic use for 2 months prior to and during the study. Methodological design in future studies should account for cross-drug tolerance in order to increase the validity of results.

#### Diagnostic continuity

While 100% of the selected studies focused on participants with a fibromyalgia diagnosis, only two studies (Van de Donk et al. 2019; Habib and Artul 2018) used established criteria to determine the diagnosis. Both studies reported using diagnostic criteria established by the American College of Rheumatology (Wolfe et al. 2010). In addition to a lack of continuity within and across studies regarding the operationalization of diagnostic parameters, one study (Habib and Avisar 2018) did not establish confirmation of a fibromyalgia diagnosis in study participants. A broader literature review indicated that precise parameters for establishing a fibromyalgia diagnosis are operationalized in widely different manners across patients, clinicians, official medical bodies, and even cultures. Diagnostic consistency was not widely established across the selected studies.

For example, Yassin et al. (2019) only included participants who had received a fibromyalgia diagnosis from an orthopedic pain clinic. Sagy et al. (2019) included patients with a confirmed diagnosis of fibromyalgia from a primary care physician but did not establish parameters for diagnostic criteria between physicians. A lack of consistent diagnostic criteria across participants reduces the strength of the internal and external validity of any study on this topic. Recommendations for future studies in this area include verification of a fibromyalgia diagnosis by using a symptoms checklist or participant inclusion criteria that are based on established and widely recognized diagnostic criteria.

In addition, the selected studies generally failed to clearly record participant symptoms at the start of each study protocol. Symptoms such as constipation, dizziness, dry mouth, and dry eyes consistent with symptoms of fibromyalgia may also be attributable to cannabis use (Van de Donk et al. 2019). Future studies should establish a baseline for symptoms that are commonly associated with fibromyalgia so as to distinguish them from the adverse effects of cannabis. Future studies should also control for cannabis use patterns when assessing adverse side effects, establishing a control group for each category of cannabis user (past uses, current user, naïve user, and non-user).

#### Comorbidities

There is a high level of agreement in the extant literature that fibromyalgia is a comorbid disorder, rarely occurring in isolation (Fitzcharles et al. 2018; Marrie et al. 2012). Fibromyalgia has been reported in up to 30% of patients with varying rheumatic conditions (Fitzcharles et al. 2018). Nearly 30% of patients with hereditary neuropathy also have fibromyalgia (Yilmaz et al. 2015), and the rate of fibromyalgia is 44% greater among individuals diagnosed with multiple sclerosis (MS) than the general population (Marrie et al. 2012). Studies have indicated that the presence and characteristics of comorbidities in part determine treatment response in fibromyalgia.

For example, the prevalence of depression in the fibromyalgia population is 25–60%, and research has shown that fibromyalgia patients with comorbid long-term or preexisting depression are less responsive to certain pain medications than fibromyalgia patients with short-term depression (Silverman et al. 2017). While two of the selected studies asked participants about comorbidities as part of the demographic questionnaire, none of the studies controlled for comorbidities or considered them during analyses. Establishing a baseline across fibromyalgia patients with diverse comorbidities is another critical aspect of methodological design, which is essential for assessing the efficacy, safety, and tolerance of cannabis for pain management. Additionally, comorbidities should be taken into consideration when establishing baselines, control groups, and reference groups.

### Outcome assessment

Several different methods were used to assess outcome across the selected studies. Van de Donk et al. (2019) assessed electrical pain thresholds and spontaneous pain scores, whereas Sagy et al. (2019) measured the overall quality of life (QOL) and degree of pain intensity. Habib and Artul (2018) and Yassin et al. (2019) assessed outcome using the Revised Fibromyalgia Impact Questionnaire (FIQR), which asks only one question regarding the level of pain. Yassin et al. (2019) also used the Patient’s Global Impression of Change (PGIC) Scale (reflects a patient’s belief about the efficacy of treatment), and the Low Back Pain (LBP) scale to assess an additional category of pain. Habib and Avisar (2018) asked questions about the effect of cannabis on pain, sleep, anxiety, and depression, but did not utilize any formal assessments.

The use of such a wide variety of assessments for determining treatment outcome, particularly concerning measuring chronic pain levels, decreases the generalizability of results across studies and the overall broader fibromyalgia patient population. Further, chronic pain as a construct was not operationalized in any of the five selected studies. Continuity regarding pain as a construct will help researchers to determine appropriate assessment measures. Operationalizing the specific type of pain that is being targeted in fibromyalgia patients in relation to cannabis as an analgesic and selecting appropriate outcome measures will be an important aspect of future studies.

### Conclusions

The authors of the selected studies collectively suggest that medical cannabis is a safe and effective treatment option for patients with fibromyalgia, with reports of significant improvements in pain intensity/severity (Habib and Artul 2018; Habib and Avisar 2018; Sagy et al. 2019; Yassin et al. 2019); sleep quality (Habib and Artul 2018; Habib and Avisar 2018; Sagy et al. 2019); level of depression (Habib and Artul 2018; Habib and Avisar 2018); level of anxiety (Habib and Artul 2018; Habib and Avisar 2018); and overall quality of life (Sagy et al. 2019).

### Additional limitations in study design across selected studies

Additional weaknesses in methodological design occurred in varying configurations across all selected studies. These weaknesses affected the overall generalizability of outcomes and included problems with inclusion and recruitment criteria, lack of control groups or appropriate reference groups, short treatment duration, and small sample sizes. Randomized clinical trials with proven methodological design are a critical need in the field of cannabis research as it pertains to assessing chronic pain management in fibromyalgia patients. The growing legalization and increasing use of cannabis across all populations indicates that cannabis use, for any reason, is a significant public health concern, and empirically-based information is urgently needed.

## Conclusion and recommendations

Although the five critically reviewed studies would seem to suggest that medical cannabis is a safe and effective treatment option for patients with fibromyalgia, the serious methodological limitations of this research preclude drawing any strong conclusions about efficacy. Instead, we advise that the reviewed body of literature lends very little evidence in support of medical cannabis as an efficacious treatment modality for chronic pain management in fibromyalgia patients. We conclude that no studies to date have established a compelling relationship between any form of medical cannabis treatment and symptom improvement in fibromyalgia patients suffering from chronic pain.

Moreover, the studies reviewed in this paper indicate a high prevalence of adverse side effects associated with cannabis use in fibromyalgia patients. The majority of reviewed studies utilized or indicated smoking as the ROA for cannabis treatment despite empirical evidence indicating that smoking cannabis has adverse health effects and is not recommended for treating chronic conditions. Randomized clinical trials using ROAs specifically indicated for increased safety, tolerance, and efficacy are needed before cannabis can be safely recommended as a treatment modality for managing chronic pain in fibromyalgia patients. It should be noted, however, that the validity of randomized clinical trials of cannabis use may be compromised by challenges to adequate participant blinding. Participants’ awareness of the lack of psychoactive effects of placebo cannabis may result in the inadvertent overestimation of the effectiveness of medical cannabis (Casarett 2018; Russo 2016).

Additionally, further research is needed in order to ascertain the clinical benefits as well as the safety and tolerability profiles of all strains and compositions of cannabis used for symptom relief. Studies are also needed to identify the effects of short- and long-term drug interactions with cannabis in fibromyalgia patients who concurrently use conventional analgesics or unauthorized forms of cannabis for chronic pain. Furthermore, fibromyalgia patients often have multiple comorbidities, making the effects of medical cannabis on specific symptoms hard to parse out across the varying symptoms brought by diverse and often overlapping medical conditions. Future study design should also include post hoc analysis to assess the effect of baseline characteristics on the mean pain scores across patients with varying baseline characteristics such as demographic specifications, comorbidities, past cannabis use, concurrent drug use, symptoms, pain levels, and other such relevant factors. Separating fibromyalgia patients into groups based on baseline indications and comparing them to reference groups is a crucial aspect of sound methodological design that was not sufficiently implemented across the selected studies in this review.

Due to the current legal climate regarding cannabis use in the United States, health professionals are often confronted with the need to educate patients regarding safe cannabinoid use. Standardization of treatment compounds and regimens is needed so that health practitioners can offer safe, evidence-based information to patients. However, given the inconsistent results across studies, reaching a definitive conclusion regarding the use of cannabinoids for chronic pain management in fibromyalgia patients is not possible at this time.

## Data Availability

Not applicable.

## Abbreviations

RCTs: Randomized clinical trials
THC: -Δ-9-tetrahydrocannabinol
CBD: Cannabidiol
ROA: Route of administration
QOL: Quality of life
FIQR: Revised Fibromyalgia Impact Questionnaire
PGIC: Patient’s Global Impression of Change
LBP: Low back pain
